# Users’ Needs for Mental Health Apps: Quality Evaluation Using the User Version of the Mobile Application Rating Scale

**DOI:** 10.2196/64622

**Published:** 2025-07-04

**Authors:** Siyeon Ko, Hyekyung Woo

**Affiliations:** 1 Department of Health Administration, College of Nursing & Health, Kongju National University, 56 Gongjudaehak-ro, Gongju-Si, Chungcheongnam-do, Republic of Korea, 82 10-3350-3486; 2 Institute of Health and Environment, Kongju National University, Gongju-si, Chungcheongnam-do, Republic of Korea

**Keywords:** app user perspective, user evaluation, mental health care, digital health, mobile app, mHealth, mental health, app, evaluation study, mobile health, quality, user, user perspective, Google Play Store, correlation analysis, regression analysis, technology, generative AI, app quality, smartphone

## Abstract

**Background:**

Mental health is an essential element of life. However, existing mental health services face challenges in utilization due to issues such as societal prejudices and a shortage of counselors. Mobile health is gaining attention as an alternative approach to improving mental health by addressing the shortcomings of traditional services. As a result, various mental health apps are being developed, but there is a lack of evaluation research on whether these apps meet users’ needs.

**Objective:**

This study aims to evaluate the content and quality of mental health apps from the user’s perspective and identify the content features that influence evaluation scores. We also aim to guide future updates and improvements in mental health apps to deliver high-quality solutions to users.

**Methods:**

We searched the Google Play Store and iOS App Store using Korean keywords “mental health,” “mental health care,” “depression,” and “stress.” Apps meeting the following criteria were selected for the study: relevance to the topic, written in Korean, more than 700 reviews (Android) or more than 200 reviews (iOS), updated within the past 365 days, available for free, nonduplicate, and currently operational. After identifying and defining the primary contents of the apps, 7 users evaluated their quality using the user version of the Mobile Application Rating Scale (uMARS). Correlation analysis was performed to examine the relationships among app content, uMARS scores, star ratings, and the number of reviews. Multiple regression analysis was conducted to identify the factors influencing uMARS scores and each evaluation item.

**Results:**

The analysis included a total of 41 mental health apps. Content analysis revealed that reminders (n=29, 71%), recording and statistics features (n=29, 71%), and diaries (n=24, 59%) were the most common app components. The top-rated apps, as determined by uMARS evaluations, consistently provided information about counselors and counseling agencies, and included counseling services. uMARS scores were significantly correlated with the presence of health care provider information (*r=*0.53; *P<*.001) and counseling/question and answer services (*r=*0.55; *P<*.001). Multiple regression analysis indicated that providing more relevant information was associated with higher uMARS scores (β*=*.361; *P=*.02).

**Conclusions:**

The quality of mental health apps was evaluated from the user’s perspective using a validated scale. To deliver a high-quality mental health app, it is essential to incorporate app technologies such as generative artificial intelligence during development and to continuously monitor app quality from the user’s perspective.

## Introduction

Mental health is an essential aspect of life, representing a positive and peaceful state that enables individuals to effectively manage daily stress [1]. Mental health problems can greatly reduce the quality of life and are often recognized as major contributors to weakened immunity and an increased risk of disease. Consequently, there is an urgent need for ongoing mental health management [2,3]. Interest in mental health care has grown markedly due to the rise in mental health issues, such as psychological stress, depression, and anxiety, exacerbated by prolonged social distancing and self-isolation during the COVID-19 pandemic [4]. Notably, Korea has the highest suicide rate among member countries of the Organization for Economic Cooperation and Development, underscoring the importance of mental health management [5].

Current mental health care and promotion services primarily focus on 1-on-1, face-to-face counseling. Consistent emotional exploration and management are key to recovering from mental health issues [6]. However, these services are often insufficient because of factors such as a shortage of counselors, inadequate facilities, financial barriers, and societal prejudices [7].

Recently, ChatGPT, a generative artificial intelligence (AI) model that generates contextually relevant responses based on user input, has gained significant popularity for its ability to reduce human intervention while enhancing efficiency. When asked about mental health care, it provides tailored solutions related to treatment, self-management, interpersonal relationships, and goal setting. This adaptive responsiveness is especially valuable for AI chatbots and other apps that require personalized services [8].

Within the field of digital health, interventions such as a “web-based tool for health promotion and stress management” and “online educational program focusing on depression and anxiety” have shown promise in addressing various mental health problems [9-11]. The proliferation of mobile health (mHealth) apps that incorporate digital health content offers an alternative approach to improving mental health, whether through face-to-face or remote interactions, while also addressing the limitations of traditional services.

mHealth apps for mental health promotion target a range of issues, including anxiety, depression, and stress. They provide information to support self-diagnosis, treatment options, and access to counseling services. The inclusion of features such as health data collection and personalized feedback helps foster user engagement and supports mental health improvement [12]. These apps have been well-received, as they compensate for the limitations of the existing services by offering a discreet, low-risk alternative that helps mitigate barriers to psychiatric counseling [13].

The growing interest in mHealth for mental health has led various institutions and developers to create apps featuring psychological support programs, mental health assessments, counseling, and management tools. However, limited awareness of these apps and the proliferation of similar apps with nearly identical functions pose challenges for users in selecting customized solutions [4,14].

Accordingly, research on the quality evaluation of mental health apps is actively underway in many countries. Although such evaluations exist, they often emphasize expert perspectives, highlighting the need for more user-centered research to assess satisfaction and usability [15-17]. In Korea, research targeting specific user groups and behaviors, such as hospital employee–focused case studies and app development for anger management, is ongoing, yet a broader understanding of whether these apps meet users’ needs remains limited [18-20].

This study assessed the content and quality of mental health apps available in South Korea from the user perspective, focusing on apps from the Google Play Store and iOS App Store. We aim to guide future updates and improvements in mental health apps to deliver high-quality solutions to users.

## Methods

### App Selection

The selection process of the apps was conducted following the PRISMA (Preferred Reporting Items for Systematic Reviews and Meta-Analyses) guidelines [21]. Between September 30 and October 5, 2022, we conducted searches on both the Google Play Store and iOS App Store using Korean keywords, including “mental health,” “mental health care,” “depression,” and “stress.” These keywords were chosen based on previous studies related to mental health and mental health apps. Following this initial search, we established specific criteria for app inclusion, considering prior research findings [22-24]. As free apps are generally more accessible to users and tend to be the most widely used, free downloads were included as a selection criterion [15]. The inclusion criteria for apps selected for the final analysis required that all of the following conditions (C1-C7) be satisfied:

- C1. Relevance to the topic
- C2. App language is Korean
- C3. App has received over 700 reviews on Android and over 200 reviews on iOS (calculated in proportion to each platform’s market share in Korea as of Q2 2022 [25])
- C4. The app was updated within the last 365 days
- C5. The app is free to use (including those with in-app purchases)
- C6. The app is nonduplicate
- C7. The app is currently operational

The star ratings for each app were extracted from the respective app stores as of the search date. On both platforms, star ratings are based on a 5-point scale. The review counts represent the cumulative total number of user reviews recorded for each app up to the search date.

### Analysis of App Contents

We downloaded all selected research apps and categorized their main contents in Multimedia Appendix 1, classifying them into categories, such as information provision, self-diagnosis, and monitoring functions. A frequency analysis was performed to determine the number of apps offering these functions.

### Evaluation of App Quality

For each of the final selected apps, we assessed quality using the Korean-translated version of the user version of the Mobile Application Rating Scale (uMARS) [26]. uMARS is a 5-point scale developed from the original Mobile Application Rating Scale to enable users to evaluate mHealth apps [27,28]. The scale demonstrates excellent internal consistency and has been widely validated, confirming its reliability and validity. The scale has been successfully applied to assess various mHealth apps, including those related to weight management, rheumatic conditions, menstrual cycles, and cancer risk calculators [22,29-31]. The overall uMARS score was calculated by averaging the scores across all 5 categories: engagement, aesthetics, functionality, information, and subjective quality.

Between October 8 and October 14, 2022, a total of 7 users of real mental health apps participated in the uMARS evaluation. The number of evaluators was determined based on user-centered evaluation standards, which suggest that 5 or more users are typically sufficient to identify most usability issues. This sample size ensures diversity in user experiences and allows for reliable results [32,33]. Apps were randomly assigned to evaluators to minimize potential bias due to subjectivity. Each evaluator was instructed to use the assigned app for at least 10 minutes daily, and evaluations were conducted in a blind test format.

### Statistical Analysis

After normalizing the data, the correlations among app content, uMARS scores, star ratings, and number of reviews were analyzed using Pearson correlation analysis. To explore the relationship between objective and subjective indicators, 4 separate correlation analyses were conducted by categorizing the aforesaid data into all apps, the top 20 apps, Android apps, and iOS apps. Multiple regression analysis was performed to identify the content factors influencing uMARS scores and evaluation items. All analyses were conducted using R software (version 4.1.2; R Core Team).

## Results

### App Selection

**Figure 1.**
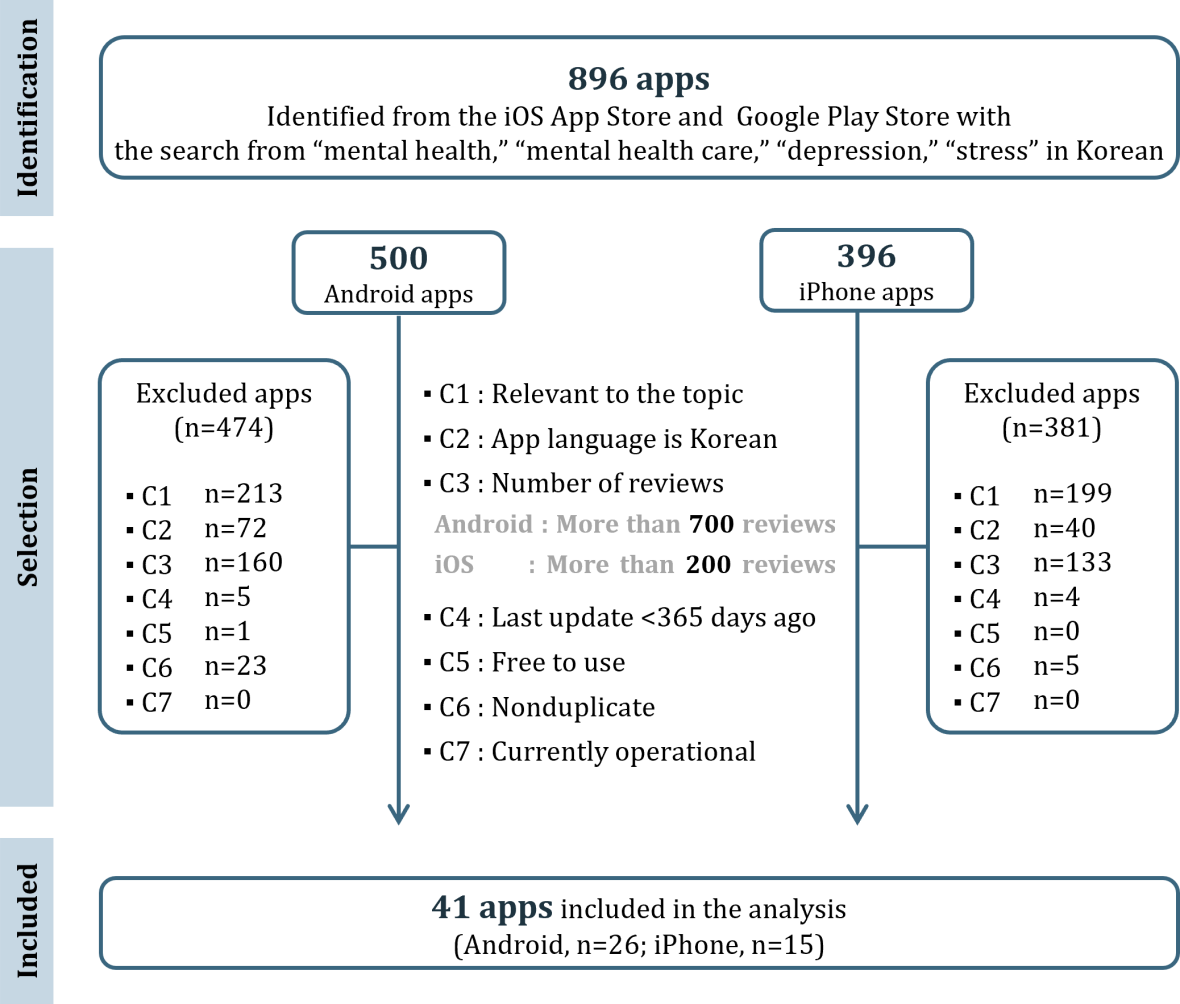
Flowchart depicting the app review and selection process (n=41). C1-7: criteria 1‐7.

A total of 896 apps were identified using the search keywords. Apps that did not meet the inclusion criteria (C1-C7) were excluded, resulting in 41 apps being selected for the final analysis. This included 26 Android apps and 15 iOS apps (Figure 1).

The app’s operating system (OS) is linked to the App Store, allowing app updates whenever the OS is updated [34]. Therefore, depending on the timing of the OS update, the version and function of each OS may differ, and the same app on different OS versions was considered a different app.

### Analysis of App Contents

Of the 41 apps, 29 (71%) included reminders, as well as recording and statistics features. Among the monitoring functions, 24 (59%) offered diary features, and 21 (51%) provided emotion tracking features. In terms of privacy, 26 (63%) included a lock feature, which was more common than in existing mHealth apps. However, only 1 (2%) provided an icon change option (Table 1).

**Table 1.** App contents analysis results.

Contents	Android (n=26), n (%)	iPhone (n=15), n (%)	Total (N=41), n (%)
Information			
Health information	9 (35)	3 (20)	12 (29)
Health care provider information	7 (27)	5 (33)	12 (29)
Cognitive behavioral therapy	8 (31)	2 (13)	10 (24)
Review	4 (15)	3 (20)	7 (17)
Function			
Reminder	18 (69)	11 (73)	29 (71)
Connected wearable devices	3 (12)	2 (13)	5 (12)
Fingerprint sensor and camera	3 (12)	1 (7)	4 (10)
Entertainment	3 (12)	1 (7)	4 (10)
Social networking site log-in interlock	15 (58)	10 (67)	25 (61)
Direct log-in	8 (31)	7 (47)	15 (37)
Service			
Mindfulness and meditation	11 (42)	7 (47)	18 (44)
Counseling/question and answer	8 (31)	6 (40)	14 (34)
Recording and statistics	20 (77)	9 (60)	29 (71)
Chatting based on algorithm	6 (23)	5 (33)	11 (27)
Self-diagnosis			
Self-diagnosis through questionnaire	10 (38)	5 (33)	15 (37)
Mood checking via artificial intelligence	2 (8)	2 (13)	4 (10)
Monitoring			
Tracker anxiety	4 (15)	2 (13)	6 (15)
Tracker depression	7 (27)	3 (20)	10 (24)
Tracker stress	7 (27)	3 (20)	10 (24)
Tracker emotions	12 (46)	9 (60)	21 (51)
Daily diary	14 (54)	10 (67)	24 (59)
Behavior change technology			
Goal and planning	5 (19)	2 (13)	7 (17)
Community and social networking sites share	14 (54)	7 (47)	21 (51)
Privacy			
Icon change	0 (0)	1 (7)	1 (2)
Lock	15 (58)	11 (73)	26 (63)

### Evaluation of App Quality

An evaluation of the quality using uMARS resulted in an overall mean score of 3.53 (SD 0.34). Among the apps, “Mind Cafe Lite” for Android achieved the highest score of 4.08 (SD 0.30), while “Depression Test” received the lowest score of 2.68 (SD 0.57). In the uMARS evaluation, the top 5 apps received their highest scores in the information category. They shared a common feature: providing information and counseling services from counselors and counseling agencies. However, most of these services, including counseling and meditation, required a fee.

Conversely, the bottom 5 apps received the lowest scores on subjective evaluation items. These apps offered limited or no counseling-related services commonly provided by the top 5 apps. Moreover, most of them offered only a single function, such as stress measurement or depression testing, which limited their utility for consistent mental health management and promotion (Table 2). uMARS scores of all 41 apps are presented in Multimedia Appendix 2.

**Table 2.** The 5 highest and lowest scoring apps based on the user version of the Mobile Application Rating Scale (n=41).

App	Operating system	Score, mean (SD)					
		Engagement	Functionality	Aesthetics	Information	Subjective quality	Overall score
Top 5^a^							
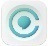	Android	3.89 (0.46)	4.43 (0.35)	4.14 (0.39)	4.43 (0.39)	3.54 (0.45)	4.08 (0.30)
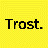	Android	4.37 (0.20)	4.11 (0.29)	4.29 (0.49)	4.29 (0.47)	3.29 (0.43)	4.07 (0.31)
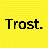	iOS	4.14 (0.33)	4.11 (0.35)	4.19 (0.35)	4.36 (0.40)	3.29 (0.60)	4.02 (0.31)
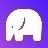	Android	3.74 (0.35)	4.36 (0.40)	4.57 (0.61)	4.46 (0.43)	2.89 (0.73)	4.01 (0.43)
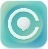	Android	3.97 (0.39)	4.21 (0.47)	4.05 (0.42)	4.32 (0.37)	3.29 (0.54)	3.97 (0.33)
Bottom 5^b^							
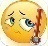	Android	2.66 (0.45)	3.54 (0.62)	2.57 (0.87)	2.96 (0.52)	1.68 (0.61)	2.68 (0.57)
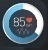	Android	2.66 (0.30)	3.57 (0.32)	2.86 (0.16)	3.32 (0.49)	1.96 (0.34)	2.87 (0.20)
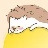	Android	2.86 (0.21)	3.32 (0.49)	3.29 (0.93)	3.18 (0.69)	2.21 (0.49)	2.97 (0.48)
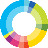	iOS	3.09 (0.50)	3.14 (0.80)	3.48 (0.56)	3.14 (0.80)	2.39 (0.72)	2.98 (0.49)
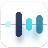	Android	2.26 (0.38)	4.18 (0.42)	3.14 (0.39)	3.64 (0.77)	2.18 (0.68)	3.08 (0.37)

a Top 5 apps: 1. Mind Cafe Lite, 2. Trost, 3. Trost, 4. Elephants – Sleep, Meditation, 5. Mind Cafe.

b Bottom 5 apps: 1. Depression Test, 2. Instantaneous Heartbeat Monitor, Health, Home Training, Stress Measurement, 3. Dochi - My Own Emotional Diary, 4. Owaves, 5. eMoods.

The top 5 apps commonly provided information about health care providers, while 3 of the apps included content related to mental health and cognitive behavioral therapy. One app offered counseling and meditation services, but these were available only through paid features such as in-app purchases. By contrast, among the bottom 5 apps, none provided information about health care providers, and only 1 offered mental health information. Most of the bottom 5 apps had single functions, such as stress measurement, depression testing, or emotional record-keeping, making them less suitable for ongoing mental health management and promotion (Multimedia Appendix 3).

### Statistical Analysis

Table 3 shows the correlations among the content of mental health apps, uMARS scores, star ratings, and number of reviews. Health care provider information (*r*=0.53; *P*<.001) and counseling/question and answer (Q&A; *r*=0.55; *P*<.001) had the strongest correlations with the uMARS score. Mindfulness and meditation (*r*=0.40; *P*=.01) and chatting algorithms (*r*=0.34; *P*=.03) showed weak correlations with it. Star rating was not correlated with app content. Recording and statistics (*r*=0.41; *P*=.009) and community and social networking site sharing (*r*=0.33; *P*=.04) showed weak correlations with the number of reviews.

**Table 3.** Correlation analysis of uMARS^a^ scores, star rating, number of reviews, and app content. Pearson *r* values are presented.

App contents	uMARS score	Star rating	Number of reviews
Information			
Health information	0.20	–0.05	0.30
Health care provider information	0.53^b^	0.03	–0.11
Cognitive behavioral therapy	0.29	0.16	0.04
Review	0.37^c^	0.06	–0.06
Function			
Reminder	0.11	0.26	–0.05
Connected wearable devices	–0.13	–0.18	0.14
Fingerprint sensor and camera	–0.05	–0.21	0.23
Entertainment	0.17	0.06	0.08
Social networking site log-in interlock	0.36^c^	0.00	–0.05
Direct log-in	0.19	–0.16	0.11
Service			
Mindfulness and meditation	0.40^c^	0.20	0.08
Counseling/question and answer	0.55^b^	0.10	–0.20
Recording and statistics	0.00	0.10	0.41^d^
Chatting based on algorithm	0.34^c^	0.05	–0.12
Self-diagnosis			
Self-diagnosis through questionnaire	0.15	–0.11	–0.05
Mood checking via artificial intelligence	0.14	–0.03	0.10
Monitoring			
Tracker anxiety	0.00	0.12	0.12
Tracker depression	–0.01	–0.05	0.17
Tracker stress	–0.01	–0.05	0.17
Tracker emotions	0.17	0.20	–0.01
Daily diary	0.33^c^	0.26	0.13
Behavior change technology			
Goal and planning	0.20	0.09	0.29
Community and social networking site share	0.25	0.20	0.33^c^
Privacy			
Icon change	–0.26	0.05	–0.24
Lock	0.30	0.22	–0.07

a uMARS: user version of the Mobile Application Rating Scale.

b *P*<.001.

c *P*<.05.

d *P*<.01.

**Figure 2.**
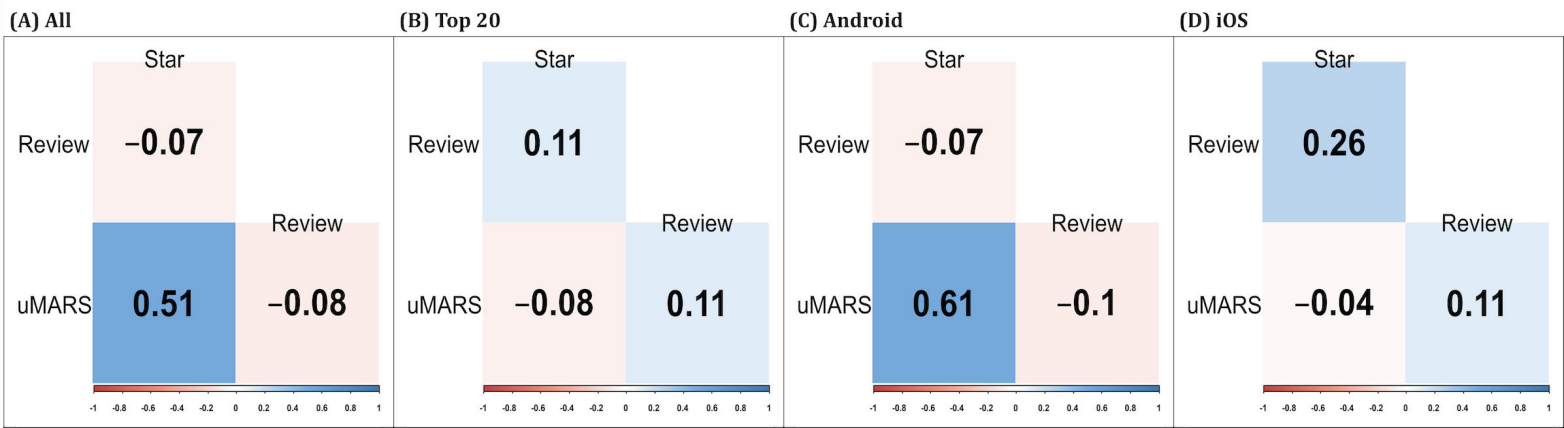
Correlation analysis for the user version of the Mobile Application Rating Scale (uMARS), star ratings, and number of reviews.

Figure 2 depicts the correlation between users’ objective uMARS score, subjective star ratings, and number of reviews. Among all apps (*r*=0.51; *P*<.001) and Android apps (*r*=0.61; *P*<.001), there was a significant correlation between the uMARS scores and star ratings. Meanwhile, no significant correlation was found between uMARS score and star ratings among the top 20 apps (*r*=–0.08; *P*=.74) and iOS apps (*r*=–0.04; *P*=.89).

Table 4 presents the outcomes of the multiple regression analysis used to identify the factors influencing uMARS scores. A higher presence of information content in an app (β=.56; *P*<.001) and a higher star rating (β=.36; *P*=.02) were associated with higher uMARS scores. Furthermore, we examined the factors influencing each evaluation item within the uMARS assessment. The presence of information content significantly influenced the overall uMARS overall score. Moreover, apps with higher star ratings had correspondingly higher scores in the aesthetics category (β=.12; *P*<.001) and the subjective evaluation category (β=.34; *P*=.04; Table 5).

**Table 4.** Multiple regression of user version of the Mobile Application Rating Scale scores.^a^

Variables	Regression		β coefficient	*t* test (*df*)
	B	SE		
Intercept	2.29	0.44	0	5.225 (30)^b^
Information	0.63	0.20	.56	3.134 (30)^c^
Functions	–0.19	0.25	–.13	–0.761 (30)
Service	0.19	0.18	.17	1.041 (30)
Self-diagnosis	–0.06	0.19	–.06	–0.323 (30)
Monitoring	–0.01	0.15	–.01	–0.058 (30)
Behavior change technology	–0.05	0.17	–.06	–0.306 (30)
Privacy	0.15	0.19	.11	0.785 (30)
Star rating	0.24	0.09	.36	2.408 (30)^d^
Number of reviews	0	0	.06	0.380 (30)
Last update	0	0	–.05	–0.333 (30)

a Adjusted *R*²=0.40, *F*_10,30_=3.68 (*P*<.001).

b *P*<.001.

c *P*<.01.

d *P*<.05.

**Table 5.** Multiple regression for the user version of the Mobile Application Rating Scale items.

Variables	Regression		β coefficient	*t* test (*df*)	Adjusted *R*²/*F* test (*df*); *P* value
	B	SE	β		
Engagement					0.37/3.36 (10, 30); <.001
Intercept	2.72	0.58	0	5.23 (30)^a^	
Information	0.60	0.27	.42	3.13 (30)^b^	
Functions	0.07	0.33	.04	–0.76 (30)	
Service	0.26	0.24	.18	1.041 (30)	
Self-diagnosis	–0.12	0.26	–.08	–0.32 (30)	
Monitoring	–0.10	0.20	–.08	–0.06 (30)	
Behavior change technology	–0.04	0.22	–.03	–0.31 (30)	
Privacy	0.51	0.25	.28	0.79 (30)	
Star rating	0.09	0.13	.11	2.41 (30)	
Number of reviews	0	0	–.07	0.38 (30)	
Last update	0	0	–.21	–0.33 (30)	
Functionality					0.24/2.28 (10, 30); <.001
Intercept	3.20	0	.46	6.92 (30)^a^	
Information	0.14	0.14	.21	0.69 (30)	
Functions	–0.82	–0.62	.26	–3.12 (30)^c^	
Service	0.28	0.26	.19	1.44 (30)	
Self-diagnosis	0.16	0.16	.20	0.79 (30)	
Monitoring	0.05	0.05	.16	0.31 (30)	
Behavior change technology	0.05	0.06	.18	0.30 (30)	
Privacy	0.12	0.09	.20	0.60 (30)	
Star rating	0.18	0.29	.10	1.72 (30)	
Number of reviews	0	0.12	0	0.66 (30)	
Last update	0	0.20	0	1.26 (30)	
Information					0.47/4.61 (10, 30); <.001
Intercept	2.69	0.00	.50	5.34 (30)^a^	
Information	1.12	0.82	.23	4.89 (30)^a^	
Functions	–0.22	–0.13	.29	–0.78 (30)	
Service	0.07	0.05	.21	0.32 (30)	
Self-diagnosis	–0.16	–0.12	.22	–0.73 (30)	
Monitoring	0.18	0.15	.17	1.02 (30)	
Behavior change technology	–0.16	–0.14	.19	–0.82 (30)	
Privacy	–0.09	–0.05	.22	–0.43 (30)	
Star rating	0.18	0.22	.11	1.54 (30)	
Number of reviews	0	0.07	0	0.48 (30)	
Last update	0	–0.04	0	–0.31 (30)	
Aesthetics					0.36/3.28 (10, 30); <.001
Intercept	1.81	0	.54	3.35 (30)^a^	
Information	0.55	0.41	.25	2.21 (30)^b^	
Functions	0.13	0.07	.31	0.41 (30)	
Service	0.04	0.03	.22	0.20 (30)	
Self-diagnosis	–0.24	–0.19	.24	–1.02 (30)	
Monitoring	0.03	0.02	.19	0.15 (30)	
Behavior change technology	–0.11	–0.09	.21	0.51 (30)	
Privacy	–0.09	–0.05	.24	–0.38 (30)	
Star rating	0.44	0.55	.12	3.56 (30)^c^	
Number of reviews	0	0.03	0	0.18 (30)	
Last update	0	–0.10	0	–0.71 (30)	
Subjective quality					0.34/3.05 (10, 30); <.001
Intercept	0.99	0	.65	1.54 (30)	
Information	0.67	0.43	.29	2.26 (30)^b^	
Functions	0.05	0.03	.37	0.14 (30)	
Service	0.29	0.18	.27	1.08 (30)	
Self-diagnosis	–0.02	–0.01	.29	–0.06 (30)	
Monitoring	–0.22	–0.16	.22	–0.99 (30)	
Behavior change technology	0.03	0.02	.25	0.13 (30)	
Privacy	0.36	0.18	.28	1.28 (30)	
Star rating	0.31	0.34	.15	2.14 (30)^b^	
Number of reviews	0	0.06	0	0.35 (30)	
Last update	0	0.02	0	0.11 (30)	

a *P*<.001.

b *P*<.05.

c *P*<.01.

## Discussion

### Principal Findings and Comparison With Prior Work

The development of various mental health apps is on the rise, and the use of mHealth apps is becoming increasingly vital in the mental health field. Nevertheless, few studies have assessed user satisfaction and app quality in the context of mental health apps. In this study, we used uMARS to evaluate mental health apps from the user perspective and to identify factors that influence user evaluations. Users preferred apps that provided information related to mental health promotion, including details about counselors and counseling institutions.

However, only a limited number of apps in our final selection provided this type of information. Mental health apps are effective in enhancing patient knowledge and symptom management, primarily due to their multimedia and audiovisual content, which can deliver mental health information and education [35,36]. Users generally perceive online health information as reliable, fostering trust in their treatment regimens and encouraging active participation in disease management [37]. Moreover, the inclusion of mental health information within these apps can contribute to improved mental health literacy among users [38]. To effectively support users in promoting their mental health through apps, future app development and updates should prioritize the inclusion of mental health–related content.

Counseling and meditation services, which constituted the primary content of the top-rated apps in user evaluations, were mostly provided for a fee. Although these apps can be downloaded for free, they often require payment for in-app purchases or access beyond a limited free trial period. The cost factor remains a prominent concern among mental health app users, and apps offering paid services may be less likely to be used [15]. Users generally prefer free apps that support independent management and monitoring of their mental health [39]. In Korea, public institutions are developing various mental health management apps; 58 of the 896 (6.47%) apps in our initial search were developed by public institutions. However, these apps were not included in our final analysis due to their extended update cycles, limited utilization, and small number of user reviews. Furthermore, mental health–related apps developed as part of research projects often focus on study objectives rather than user needs [35]. Evidence-based mental health promotion projects in the public sector are also relatively rare. To enhance app quality and encourage the use of public apps for mental health promotion, it is advisable to benchmark the services offered by top-ranking paid apps and align them with research-driven initiatives.

Chatting based on algorithms demonstrated a weak but significant correlation with uMARS scores. Users are increasingly interested in utilizing AI chatbots that provide convenient access to medical information without relying solely on health care providers [40]. Emerging technologies, such as generative AI, have the potential to significantly impact the health care sector by delivering tailored health advice, support, and integrated systems for detecting and managing health issues [8,41]. In the field of mental health, generative AI can serve as a valuable tool to support the treatment process by offering functions such as 24-hour online support, counseling record maintenance, and follow-up management [42]. It also has the potential to bridge gaps in the diagnosis and treatment of mental disorders, expanding access to psychiatric care [43]. Currently, AI chatbot functions in mental health apps are somewhat limited, often restricted to depression self-diagnosis tests (eg, the 9-item Patient Health Questionnaire). Additionally, technical issues such as limited response capabilities and unnatural conversation flow have been noted. The lack of personalized feedback for continuous engagement also hinders user satisfaction and long-term participation [44-47]. If AI counseling services that provide evidence-based, customized solutions by integrating existing mental health apps with generative AI functions are developed, they are expected to deliver higher-quality mental health services to users. To achieve this, it is essential to explore future app development strategies that incorporate generative AI in diverse ways. These efforts should focus on developing advanced algorithms that enhance natural conversation flow and provide sophisticated, personalized support. Furthermore, continuous clinical validation is necessary to ensure the reliability, accuracy, and safety of AI technologies.

The correlation between the uMARS score, an objective quality evaluation tool for mHealth apps, and user star ratings differed depending on the platform. Considering previous studies reporting digital disparities and age differences between Android and iOS users [48,49], these factors may have contributed to variations in app evaluation criteria, user preferences, and expectation levels. However, some studies have found no significant differences in personality traits or characteristics between users of the 2 platforms [50]. Combining these conflicting findings, it can be inferred that differences in user evaluations across platforms may be influenced not only by user characteristics but also by app usage experiences and platform-specific features. Future research should develop evaluation strategies that systematically analyze these differences and account for platform-specific characteristics.

Mental health apps collect and share sensitive information, including user symptoms and counseling records. When handling such sensitive personal data, it is crucial to build trust with users by ensuring the protection of their personal information [51]. Many users consider features such as app locking to be essential when using mental health apps [39]; however, few of the apps included in our final study offered the option to change the app icon for privacy purposes, and some even lacked lock functionality. Moreover, concerns about unclear personal information protection, the absence of relevant regulations, and a lack of personalized attention from apps have been steadily raised in mHealth research. As a result, some countries are now beginning to implement personal data protection regulations [52-54]. While regulations such as the Health Insurance Portability and Accountability Act and the General Data Protection Regulation were developed to protect personal information, they are not universally applied, leaving privacy and security challenges unresolved [55]. In response to these challenges, researchers are actively exploring sustainable systems to safeguard personal information using blockchain technology [56]. Blockchain-based health care systems offer personal information protection and provide users the option to request the deletion of their data [57]. In the mental health field, preserving the security and confidentiality of users’ mental health data is considered paramount [58]. Consequently, app developers must prioritize including privacy features suited to the app’s characteristics and aligned with existing personal information protection regulations.

None of the apps in our final analysis targeted specific population groups. Although apps for children and adolescents are in development, they remain difficult to find, and their specific benefits for particular target groups are still unclear [59]. Mental health issues vary across life stages, implying that desired app content may also differ by age group [60]. Previous research highlights the importance of tailoring interventions to life stages due to variations in mental health problems [61,62]. However, such a life stage–specific approach has yet to be fully integrated into app development. Future efforts should focus on developing mental health apps that address the unique needs of each life stage, considering factors such as sex and age. This requires active research into the usability and effectiveness of apps across different population groups to better address the diverse needs of users.

The World Health Organization (WHO) and the Centers for Disease Control and Prevention (CDC) offer targeted guidelines for mental health management, categorized by different population groups such as parents, adults, children, individuals with mental health conditions, public health workers, and health care professionals [63,64]. WHO also provides translated stress-management guides for use in different countries, along with illustrated materials that are easy for users to follow [65]. The CDC has created a dedicated page that consolidates guidelines from various institutions and includes links to mental health experts that can be accessed during emergencies. This allows users to access free or low-cost mental health services through a structured system based on standardized criteria, enabling them to choose an expert, appointment time, and office environment. By contrast, such detailed interventions are not yet standard practice for mental health management in Korea. Consequently, mental health services are often underutilized. To promote mental health services in the future, concerted efforts are required to raise awareness and ensure easy access to services through diverse interventions, such as national guidelines and expert-led initiatives offered by the WHO and CDC.

### Limitations

This study had some limitations. First, the apps were selected based on searches conducted within app stores during a specific period. As app stores are continuously updated, the content analyzed in this study may differ from that of future app versions. Second, the data sources were not electronic academic databases but app stores, and the accuracy of the search process may have varied depending on each store’s recommendation algorithms.

### Conclusions

In this study, users evaluated the quality of mental health apps using a validated scale. Users preferred apps that included information related to mental health. To develop high-quality mental health apps, it is essential to incorporate technologies such as generative AI into the app development process. This study is meaningful in that it identified the types of content preferred by users through a quality evaluation and suggested directions for developing apps that meet current needs. We expect our results to serve as guidelines for creating evidence-based data and developing health promotion apps in the public sector. Further research focusing on the long-term usability, effectiveness, and user engagement of mental health apps—through extended evaluations from a user perspective—could contribute to improving their overall quality and impact.

## Supplementary material

10.2196/64622 Multimedia Appendix 1 Description of the app contents.

10.2196/64622 Multimedia Appendix 2 The user version of the Mobile Application Rating Scale score of all 41 apps.

10.2196/64622 Multimedia Appendix 3 Content comparison between the top and bottom 5 apps (ranked according to the user evaluation scores).

## Abbreviations

AI: artificial intelligence
CDC: Centers for Disease Control and Prevention
mHealth: mobile health
OS: operating system
PRISMA: Preferred Reporting Items for Systematic Reviews and Meta-Analyses
Q&A: question and answer
uMARS: user version of the Mobile Application Rating Scale
WHO: World Health Organization
