# Supplementary material for: Users’ Needs for Mental Health Apps: Quality Evaluation Using the User Version of the Mobile Application Rating Scale
Source: JMIR Mhealth Uhealth. 2025 Jul 4;13:e64622. doi: 10.2196/64622 (PMC12248136; doi:10.2196/64622)
Supplement: Multimedia Appendix 1 [file mhealth-v13-e64622-s001.docx]

Table 1. App contents description

| **Contents** | | **Label** | **Description** |
| --- | --- | --- | --- |
| **Information** | |  |  |
|  | Health information | Providing health-related information | Provides a variety of information related to mental health |
|  | Health care provider information | Provide counselor and counseling institutions with information | Providing information, such as history of counselors, available counseling period, counseling institutions, and methods of application for counseling, and so forth |
|  | Cognitive behavioral therapy | Provide cognitive behavior therapy and technical information | Provide relevant information, such as controlling the role and behavior of therapists |
|  | Review | Counselor review information | Review information on counselors and psychological counseling reviews to confirm the effectiveness of counseling |
| **Function** | |  |  |
|  | Reminder | Notification function | Reminders to use the app (push alerts, and notifications for sound and screen activation) |
|  | Connected wearable devices | Wearable interlocking function | Wearable interlocking function |
|  | Fingerprint sensor and Camera | Measurement of health conditions such as stress and heart rate changes using fingerprint sensors and cameras | Measurement of health conditions such as stress and heart rate changes using fingerprint sensors and cameras |
|  | Entertainment | Gaming and entertainment function | Funn and interesting elements of a functional game |
|  | SNS log-in interlock | Integrated login method using SNS login linkage | Integrated login method using SNS login linkage |
|  | Direct log-in | Create your own ID and password and log in | Create your own ID and password and log in |
| **Service** | |  |  |
|  | Mindfulness and meditation | Providing meditation services | Provides stress management services such as mindfulness and meditation |
|  | Counseling / Q&A | Providing counseling services | Providing counseling with psychological counselors and question-and-answer services |
|  | Recording and statistics | Records and statistics | Records of personal health information and provision of weekly and monthly statistical services |
|  | Chatting based on algorithm | Algorithm-based chat functionality | Provides real-time chat and consultation solutions using AI |
| **Self-diagnosis** | |  |  |
|  | Self-diagnosis through questionnaire | Self-diagnosis through questionnaires | Self-diagnosis through questionnaires |
|  | Mood checking via AI | Checking mental status using AI chatbot | Checking mental status using AI chatbot |
| **Monitoring** | |  |  |
|  | Tracker anxiety | Tracking and monitoring anxiety | Provides real-time anxiety tracking analysis and feedback |
|  | Tracker depression | Tracking and monitoring depression | Provides real-time depression tracking analysis and feedback |
|  | Tracker stress | Tracking and monitoring stress | Provides real-time stress tracking analysis and feedback |
|  | Tracker emotions | Tracking and monitoring emotions | Provides real-time emotion tracking analysis and feedback |
|  | Daily diary | My daily life check | Provides management services such as diaries and personal daily records for mindfulness |
| **Behavior Change Technology** | |  |  |
|  | Goal and planning | Set goals | Sets goals for behavior change and provide motivation to achieve them |
|  | Community & SNS share | Community and sharing | Communities and social media sharing functions that allow communication among users |
| **privacy** | |  |  |
|  | Icon change | App icon change for privacy | Icon changes that make it seem unrelated to the mental health app |
|  | Lock | Password setting for the app | Setting and locking passwords for the protection of consultation information and personal information in the app |
