# Supplementary material for: Users’ Needs for Mental Health Apps: Quality Evaluation Using the User Version of the Mobile Application Rating Scale
Source: JMIR Mhealth Uhealth. 2025 Jul 4;13:e64622. doi: 10.2196/64622 (PMC12248136; doi:10.2196/64622)
Supplement: Multimedia Appendix 3 [file mhealth-v13-e64622-s003.docx]

Table 1. Content comparison between the top and bottom five apps (ranked according to the user evaluation scores).

| **Content** | | **Top 5 app** | | | | | **Bottom 5 app** | | | | | |
| --- | --- | --- | --- | --- | --- | --- | --- | --- | --- | --- | --- | --- |
|  |  | **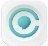** | **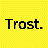** | **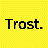** | **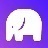** | **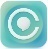** | **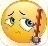** | **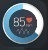** | **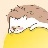** | **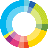** | **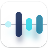** |  |
| **Information** | |  |  |  |  |  |  |  |  |  |  |  |
|  | Health information |  | ● | ● | ● |  |  | ● |  |  |  |  |
|  | Health care provider information | ● | ● | ● | ● | ● |  |  |  |  |  |  |
|  | Cognitive behavioral therapy |  | ● | ● | ● |  |  |  |  |  |  |  |
|  | Review | ● |  |  |  | ● |  |  |  |  |  |  |
| **Function** | |  |  |  |  |  |  |  |  |  |  |  |
|  | Reminder |  | ● | ● | ● |  |  |  | ● | ● | ● |  |
|  | Connected wearable devices |  |  |  |  |  |  | ● |  |  |  |  |
|  | Fingerprint sensor & Camera |  |  |  |  |  |  | ● |  |  |  |  |
|  | Entertainment |  |  |  |  |  |  |  |  |  |  |  |
|  | SNS log-in interlock | ● | ● | ● | ● | ● |  | ● | ● | ● |  |  |
|  | Direct log-in |  | ● | ● |  | ● |  | ● |  | ● |  |  |
| **Service** | |  |  |  |  |  |  |  |  |  |  |  |
|  | Mindfulness & Meditation |  | ● | ● | ● |  |  |  |  |  |  |  |
|  | Counseling / Q&A | ● | ● | ● | ● | ● |  |  |  |  |  |  |
|  | Recording & Statistics |  |  |  |  | ● |  | ● | ● |  | ● |  |
|  | Chatting based on algorithm | ● |  |  |  | ● |  |  |  |  |  |  |
| **Self-diagnosis** | |  |  |  |  |  |  |  |  |  |  |  |
|  | Self-diagnosis through questionnaire |  | ● | ● |  | ● | ● |  |  |  | ● |  |
|  | Mood checking via AI |  |  |  |  |  |  |  |  |  |  |  |
| **Monitoring** | |  |  |  |  |  |  |  |  |  |  |  |
|  | Tracker anxiety |  |  |  |  |  |  |  |  |  | ● |  |
|  | Tracker depression |  | ● | ● |  |  |  | ● |  |  | ● |  |
|  | Tracker stress |  | ● | ● |  |  |  | ● |  |  | ● |  |
|  | Tracker emotions |  | ● | ● | ● |  |  |  | ● |  | ● |  |
|  | Daily diary |  | ● | ● | ● |  |  |  |  |  | ● |  |
| **Behavior Change Technology** | |  |  |  |  |  |  |  |  |  |  |  |
|  | Goal and planning |  |  |  |  |  |  |  |  |  |  |  |
|  | Community & SNS share | ● |  |  | ● | ● |  |  |  |  | ● |  |
| **Privacy** | |  |  |  |  |  |  |  |  |  |  |  |
|  | Icon change |  |  |  |  |  |  |  |  | ● |  |  |
|  | Lock | ● | ● | ● |  | ● |  |  | ● |  |  |  |
